# Individual Factors Contributing to Nausea in First-Time Chemotherapy Patients: A Prospective Cohort Study

**DOI:** 10.3389/fphar.2019.00410

**Published:** 2019-04-25

**Authors:** Karin Meissner, Nicola Talsky, Elisabeth Olliges, Carmen Jacob, Oliver J. Stötzer, Christoph Salat, Michael Braun, Raluca Flondor

**Affiliations:** ^1^Institute of Medical Psychology, Faculty of Medicine, LMU Munich, Munich, Germany; ^2^Division of Health Promotion, Coburg University of Applied Sciences, Coburg, Germany; ^3^Clinical Neurosciences, Clinical and Experimental Sciences, Faculty of Medicine, University of Southampton, Southampton, United Kingdom; ^4^Wessex Neurological Centre, University Hospital Southampton NHS Foundation Trust, Southampton, United Kingdom; ^5^Haematology and Oncology, Outpatient Cancer Care Center, Munich, Germany; ^6^Breast Center, Department of Gynecology, Red Cross Hospital, Munich, Germany

**Keywords:** nausea, CINV, expectation, nocebo effect, cancer, chemotherapy, quality of life

## Abstract

**Objective:**

The expectation of developing side effects can enhance the likelihood to develop them – a phenomenon referred to as nocebo effect. Whether nocebo effects can be reduced by lowering negative expectancies, is not clear. The aim of this prospective study was to learn more about the factors contributing to nausea expectancy and their potential role in actual occurrence of nausea in patients undergoing chemotherapy for the first time in their life.

**Methods:**

Patients scheduled for moderately emetogenic chemotherapeutic regimens filled in questionnaires to assess state anxiety and quality of life and to rate the expectancy of nausea as a side effect of chemotherapy. Patient diaries were used to monitor the severity of post-chemotherapy nausea in the 4 days following chemotherapy administration. Bivariate analyses complemented by multiple regression analyses were performed to identify the relationship between nausea expectation and nausea occurrence.

**Results:**

121 female patients (mean age 53 years) with completed questionnaires were included in the analyses. The majority of the patients had a diagnosis of breast cancer (86%). The two main sources for nausea expectancy were positive history of nausea in other situations and state anxiety. Patients with high expectancy levels (first quartile) experienced greater nausea than those with lower expectancy levels. Bivariate analyses revealed a weak but non-significant association between nausea expectation and post-chemotherapy nausea. When controlling for age, type of cancer, history of nausea, state and trait anxiety, and global quality of life, positive history of nausea (*OR* = 2.592; 95% CI, 1.0 to 6.67; *p* < 0.05), younger age (*OR* = 0.95; 95% CI, 0.92 to 0.99; *p* < 0.05), and a lower quality of life (*OR* = 0.97; 95% CI, 0.94 to 1.0; *p* < 0.05), but not nausea expectancy (*OR* = 1.014; 95% CI, 0.51 to 2.02; *p* = 0.969), predicted the occurrence of post-chemotherapy nausea.

**Conclusion:**

In this female cohort, younger patients with lower initial quality of life and a positive history of nausea were at higher risk to develop nausea after first time chemotherapy. These patients may benefit from psychological co-interventions that aim to enhance quality of life.

## Introduction

Besides pain and constipation, nausea and vomiting are among the most disabling physical symptoms in patients receiving chemotherapy (Breen et al., 2009). In contrast to vomiting, the treatment of nausea remains challenging (Andrews and Sanger, 2014). Several risk factors have been described for the development of post-treatment nausea, including younger age (Dodd et al., 1996; Montgomery et al., 2010; Rha et al., 2016), female sex (Lohr, 2008), history of nausea during motion or pregnancy (Roscoe et al., 2004; Lohr, 2008), higher emotional distress (Blasco et al., 2000), and lower quality of life (Colagiuri et al., 2008). In addition, the expectancy of chemotherapy-induced nausea is related to the frequency and severity of nausea as a side effect of chemotherapy (Colagiuri et al., 2008; Sohl et al., 2009; Colagiuri and Zachariae, 2010; Devlin et al., 2017; Fletcher et al., 2018). Expectancy-related side effects that cannot be attributed to the pharmacological or other active ingredients are commonly referred to as “nocebo effects” (Barsky et al., 2002).

Nocebo effects account for a significant proportion of the reported side effects of medical treatments (Rief et al., 2009; Mora et al., 2011; Häuser et al., 2012; Nestoriuc et al., 2016). For example, Mahr et al. (2017) analyzed the adverse events in 231 randomized placebo-controlled trials and concluded that most of them (77–100%) occurred also in the placebo groups and thus were non-specific in nature. Since adverse events are known to negatively affect quality of life (Colagiuri et al., 2008) and patient adherence (Kardas et al., 2013), the prevention of nocebo effects bears big potential to optimize medical care by minimizing treatment discontinuation. Therefore, it is important to learn more about the factors that contribute to nocebo effects in order to find effective approaches to prevent them.

Rief et al. (2017) could recently show that a psychological intervention designed to enhance positive treatment expectations prior to surgery led to an improvement in quality of life and fitness for work 6 months after surgery (von Blanckenburg et al., 2015; Rief et al., 2017). Similarly, it has been suggested that the reduction of negative expectations may reduce the occurrence of nocebo side effects (Nestoriuc et al., 2016; Petrie and Rief, 2018). If this is true with regard to chemotherapy-induced nausea, it is important to learn more about the factors that contribute to nausea expectancy in this patient group. Previous studies indicate a difference between first-time chemotherapy patients and patients with prior chemotherapy experience: While in chemotherapy-naïve patients higher emotional distress, higher trait anxiety, and lower quality of life were associated with greater expectation of nausea (Montgomery and Bovbjerg, 2003; Colagiuri et al., 2008), prior experience with chemotherapy-related nausea was the main predictor for nausea intensity following subsequent chemotherapy infusions (Montgomery and Bovbjerg, 2003).

However, so far it is unknown whether the link between expected and perceived nausea is causal in nature. Especially in patients with previous chemotherapy experience, the relationship might simply reflect the patients’ knowledge of being more or less susceptible to chemotherapy-induced nausea. Therefore, studies in chemotherapy-naïve patients are needed to further elucidate the relationship between expected and perceived nausea. Colagiuri et al. (2008) investigated 672 first-time chemotherapy patients and confirmed expectation as an independent predictor for post-chemotherapy nausea when controlling for demographic and psychosocial factors. Further studies of this kind, however, are needed to consolidate this finding.

We performed a prospective study to further investigate the sources of nausea expectancy and post-chemotherapy nausea in patients newly diagnosed with cancer. We focused on chemotherapeutic regimens with moderate emetogenic potential. Based on previous studies (Blasco et al., 2000; Montgomery and Bovbjerg, 2003; Colagiuri et al., 2008), we hypothesized that higher emotional distress, higher trait anxiety and lower quality of life would significantly contribute to nausea expectancy before first-time chemotherapy. In addition, we hypothesized that lower age, female sex, lower quality of life, and higher nausea expectancy (Dodd et al., 1996; Blasco et al., 2000; Roscoe et al., 2004; Colagiuri et al., 2008; Lohr, 2008; Montgomery et al., 2010; Rha et al., 2016) would be independent predictors for post-chemotherapy nausea in multiple regression analyses.

## Materials and Methods

### Patients

Patients were eligible when they were aged ≥18 years, had any cancer diagnosis, were scheduled for a chemotherapy regimen with moderate emetogenic potential (Hesketh, 1999; Roila et al., 2016), had never received chemotherapy before in their life, and had sufficient knowledge of the German language to understand the wording of the questionnaires. Patients receiving a combination of chemotherapy and radiotherapy were excluded, as radiotherapy itself may induce nausea (Roila et al., 2016). To ensure compliance with the study protocol, patients with severe psychiatric diseases, such as acute psychosis, severe uni- or bipolar disorders, or dementia, were excluded. Written informed consent was obtained from each participant. The study protocol was approved by the ethical committee of the Medical Faculty at LMU Munich, Germany (Ref. No. 259-13).

### Procedure

Consecutive recruitment took place during routine primary care at two hospitals and two oncological practices from December 2013 to March 2016 until the pre-planned sample size of 130 patients with completed questionnaires was reached. Patients fulfilling all inclusion and none of the exclusion criteria received written information on the aims and the procedure of the study. In more detail, they were informed that the aim of the study was to learn more about the individual factors influencing nausea as a side effect of chemotherapy. In addition, every patient was informed during informed consent by the attending physician about the benefits and risks of chemotherapy, including natural frequencies of the most common and most serious side-effects. They were further informed that the standard antiemetic drugs available today can significantly reduce the occurrence of severe nausea. Consented patients completed the baseline questionnaires 2 days prior to the first chemotherapy administration. At this visit, they were also given the patient diary for days 1–4 after chemotherapy.

### Measures

Health-related quality of life was assessed using the European Organization for Research and Treatment of Cancer–Core Quality of Life Questionnaire (EORTC QLQ-C30, version 3.0) (Aaronson et al., 1993; Roila et al., 2016). It comprises 30 items, which can be partly combined to scales: a global health-related quality of life scale, five functional scales (physical, role, cognitive, emotional, and social), three symptom scales (fatigue, pain, and nausea and vomiting), and a number of single items assessing additional symptoms commonly reported by cancer patients (dyspnoea, loss of appetite, insomnia, constipation, and diarrhea) and perceived financial impact of the disease. Our analysis focuses on the global scale as a generalized measure of health-related quality of life. Scores range from 0 to 100, with higher scores indicating higher QoL. State and trait anxiety were assessed by means of the Spielberger State-Trait Anxiety Inventory (STAI; Spielberger, 1983).

History of nausea and expectation of developing nausea as a result of chemotherapy were assessed using a self-constructed questionnaire. To assess the history of nausea, patients were asked if they had experienced nausea previously in their life (yes/no), for example, when traveling, after surgery, during pregnancy, or following medication. In case of a positive answer, patients were asked to specify the types of experienced nausea. Patients’ expectations for post-chemotherapy nausea were assessed by four questions, which were combined to a single expectancy score (Colagiuri et al., 2008). In a first question, patients were asked to rate the likelihood that they would experience nausea after chemotherapy on a 7-point scale ranging from 1 (“I am certain I will not experience nausea”) to 7 (“I am certain I will experience nausea”). In a second question, patients were required to rate the expected severity of their post-chemotherapy nausea as “none at all,” “very mild,” “mild,” “moderate,” “severe,” “very severe,” or “intolerable.” In a third question, the patients were asked to rate their perceived susceptibility to nausea compared with their friends and family as either “more,” “less,” or “the same.” A final question asked patients to rate the likelihood of experiencing chemotherapy-related nausea compared with other cancer patients with the same diagnosis and undergoing the same treatment as “more,” “less,” or “the same.” These 4 expectancy questions were combined to a composite expectancy measure (“nausea expectancy score”) by averaging z-scores (Colagiuri et al., 2008). In accordance with Colagiuri et al. (2008), we used quartiles of nausea expectancy scores to create four groups of expectancy, classified as “not expectant” (0–25th percentile), “slightly expectant” (26–50th percentile), “somewhat expectant” (51–75th percentile), and “highly expectant” (76–100th percentile). Further questions of the self-constructed questionnaire were assessed patients’ attitudes toward chemotherapy (results not reported). In addition, a standardized questionnaire was used to record sociodemographic and clinical information from the patients’ records.

Information on post-chemotherapy nausea was collected by means of a patient diary during the 4 days following chemotherapy according to Colagiuri and Zachariae (2010). Every evening, patients rated the severity of their nausea during the past 24 h on a 7-point scale ranging from 1 (“not at all nauseated”) to 7 (“extremely nauseated”). Mean nausea was calculated as the average over these 4 days.

### Statistical Analysis

Sample size calculation was performed for the correlation between nausea expectancy and perceived nausea. Assuming a correlation coefficient of 0.25 (Montgomery and Bovbjerg, 2003; Roscoe et al., 2010), 123 subjects would be needed to give 80% power to detect a significant difference from zero with a type 1 error (two-tailed) of 5% (Cummings and Hulley, 1988). The pre-planned sample size was set at 130 patients with completed questionnaires.

Since mean nausea scores were significantly skewed, we used non-parametric tests throughout and dichotomized the variable to represent the occurrence of nausea (presence or absence) rather than severity (Montgomery and Bovbjerg, 2003) prior to multiple regression analysis.

Linear regression analysis was performed to identify predictors of the nausea expectancy score. History of nausea in other situations (no, yes), age, diagnosis (breast cancer, other types of cancer), state and trait anxiety, and global quality of life were included as predictors. Binary logistic regression analysis was used to evaluate the impact of prior nausea experience, nausea expectancy, age, diagnosis, state anxiety, and global quality of life on the occurrence of post-treatment nausea. All analyses were conducted using SPSS software (IBM, version 24). Results were considered significant when *p* < 0.05.

## Results

### Patient Characteristics

**FIGURE 1:**
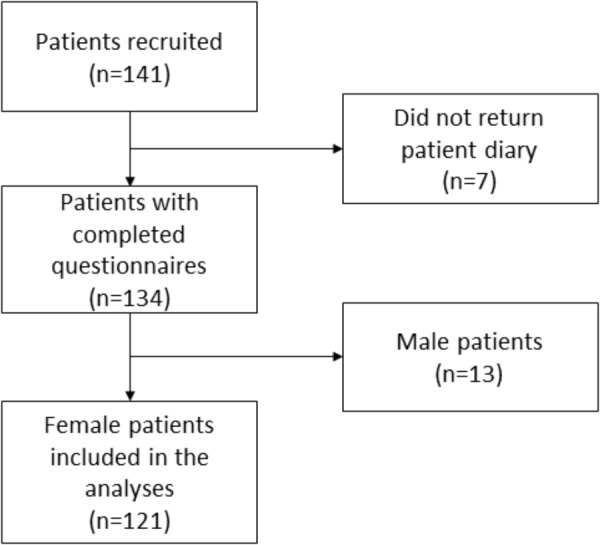
Flow chart.

141 patients were recruited and completed the baseline questionnaires, of whom 134 patients returned the patient diary. Since the cohort comprised only 13 male patients, we restricted the final analyses to female patients (*n* = 121; Figure 1). Sociodemographic and clinical patient characteristics are shown in Table 1. At baseline, 51 out of 121 patients (42%) indicated to have experienced nausea in other situations, most frequently associated with motion sickness, pregnancy, and/or surgery (Table 1).

**Table 1:** Sample characteristics at baseline.

Patient characteristics (*n* = 121)		
Age, mean (*SD*), range		53 (12), 25-84
Married/having partner, *n* (%)		102 (84)
Children, *n* (%)		90 (75)
Education, high school, *n* (%)		70 (58)
Type of cancer, *n* (%)		
	Breast cancer	104 (86)
	Ovarian or uterine cancer	8 (7)
	Gastrointestinal cancer	4 (3)
	Lymphoma	3 (3)
	Other	2 (2)
Prior nausea experience, *n* (%)		
	None indicated	70 (58)
	Motion sickness-related nausea	31 (26)
	Pregnancy-related nausea	17 (14)
	Postoperative nausea and vomiting	14 (12)
	Migraine-related nausea	5 (4)
	Nausea as side effect of medical treatment	3 (3)
STAI State-Trait-Anxiety Scale, mean (*SD*)		
	State anxiety	39.5 (10.3)
	Trait anxiety	39.8 (7.8)
Quality of life (EORCT-QLQ-C30), mean (*SD*)		
	Global QoL	64.9 (21.1)
	Physical functioning	82.0 (17.5)
	Role functioning	59.0 (30.3)
	Emotional functioning	61.0 (24.5)
	Cognitive functioning	78.5 (22.7)
	Social functioning	69.7 (25.5)

The majority of patients (*n* = 93, 77%) received cyclophosphamide (<1500 mg/m^2^) in combination with further antineoplastic agents (typically epirubicin; *n* = 85, 70%), 22 patients (18%) received platin-based chemotherapeutic regimens, and 5 patients were administered either doxorubicin or epirubicin as monotherapy (4%). All patients received a standard regimen of i.v. antiemetic medications at time of chemotherapy infusion (i.e., ondansetron and dexamethasone), supplemented by antiemetic prophylaxis for delayed nausea during the 4 days after chemotherapy including one or more of the following: metoclopramid, omeprazol, aprepitant, dimenhydrinat, and/or ondansetron.

### Expectancy of Nausea

Thirty-one out of 121 patients were classified as highly expectant of experiencing nausea as a side effect of chemotherapy, and 30 patients as somewhat expectant, slightly expectant, and not expectant, respectively. Patients with prior experience of nausea in other situations were significantly more likely to expect higher levels of nausea than patients without (χ^2^ = 10.671, *p* = 0.014) (Table 2).

**Table 2:** Levels of nausea expectancy in first-chemotherapy patients with and without prior experience of nausea in other situations.

		Not expectant	Slightly expectant	Somewhat expectant	Highly expectant	*P*-value (χ^2^ test)
Prior experience with nausea in other situations						0.019
	yes (*n* = 51)	8 (16%)	9 (18%)	16 (31%)	18 (35%)	
	no (*n* = 70)	23 (33%)	20 (29%)	15 (21%)	12 (17%)	

Linear regression analysis was performed to investigate the impact of prior nausea experience, age, type of cancer, state and trait anxiety, and global quality of life on nausea expectancy (Table 3). The overall model was significant and accounted for 28% of the variance (*R*^2^ = 0.28, *F*_(6,118)_ = 7.1, *p* < 0.001). Significant predictors for nausea expectancy were prior nausea experience (*p* < 0.001) and state anxiety (*p* = 0.005), with beta coefficients of β = 0.32 and β = 0.37, respectively (Table 3).

**Table 3:** Linear regression analysis for nausea expectancy scores.

Predictor	b	SE	β	*P*-value
Nausea in other situations (no^∗^/yes)	0.502	0.128	0.327	<0.001
Age	−0.001	0.005	−0.017	0.843
Diagnosis (breast cancer^∗^/other cancer)	0.193	0.190	0.089	0.311
State anxiety (STAI-State)	0.024	0.008	0.320	0.005
Trait anxiety (STAI-Trait)	0.016	0.010	0.165	0.103
Global quality of life (EORTC-QLQ-C30)	0.003	0.004	0.080	0.425

*SE, standard error; EORTC-QLQ-C30, European Organization for Research and Treatment of Cancer – Quality of Life Questionnaire-C30; STAI, State-Trait-Anxiety Inventory. ^∗^reference category.*

### Post-chemotherapy Nausea

During the 4 days after the first dose of chemotherapy, 72 patients (60%) suffered from at least mild nausea and 7 patients (5%) reported strong nausea (≥6 on 7-point scale); 42 patients (35%) did not report post-treatment nausea. Mean nausea equaled 1.9 out of 7 points (1.0 SD).

Mean nausea scores differed significantly between the four expectancy levels, with highest values observed in the “highly expectant” group (χ^2^ = 8.269, *p* = 0.041; Table 4). Bonferroni-corrected *post hoc* tests indicated significantly higher nausea in the “highly expectant” group compared to the “somewhat expectant” group (*U* = 275, *p* = 0.027), but no significant difference to the “slightly expectant” group (*U* = 272, *p* = 0.075) and to the “no expectant” group (*U* = 348, *p* = 0.303). When dichotomizing expectancy levels into “highly expectant” and all other expectancy levels, nausea differed significantly between groups (*U* = 928, *p* = 0.007; Table 4). The correlation between nausea expectancy scores and mean nausea did not reach the level of significance (Spearman’s rho = 0.125, *p* = 0.170).

**Table 4:** Mean nausea scores during the 4 days after chemotherapy in patients with different levels of nausea expectancy.

	Not expectant	Slightly expectant	Somewhat expectant	Highly expectant	*P*-value
Mean nausea, mean (*SD*)	1.8 (1.0)	1.7 (0.8)	1.7 (1.2)	2.3 (1.1)	0.041^∗^
			
Mean nausea, mean (*SD*)	1.8 (1.0)			2.3 (1.1)	0.007^#^

*^∗^Kruskal-Wallis-test, ^#^Mann-Whitney-U test.*

**FIGURE 2:**
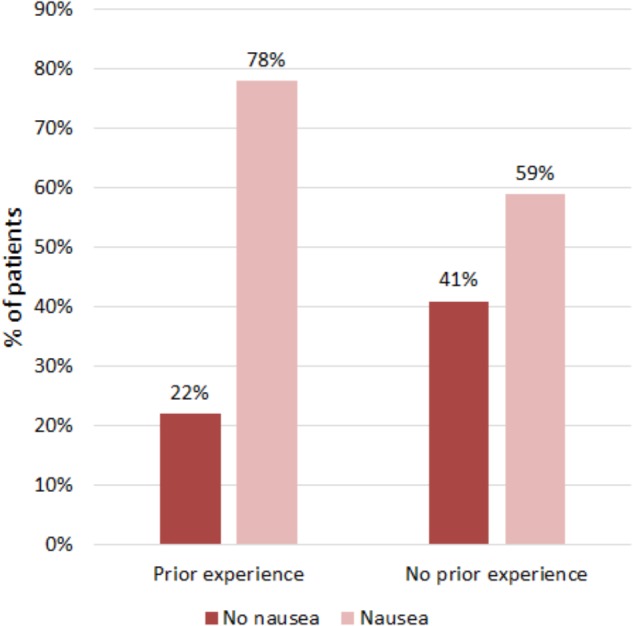
Occurrence of nausea in the 4 days after chemotherapy in patients with and without prior experience of nausea in other situations.

Patients, who had experienced nausea in other situations, were at higher risk of developing nausea after chemotherapy (40 out of 51, 78%) than patients without such experience (41 out of 70; 59%; χ^2^ = 5.259, *p* = 0.022; Figure 2). Similarly, mean nausea was significantly higher in patients with prior nausea experience (2.2 ± 1.1 SD) than in those without (1.7 ± 0.9 SD; *U* = 1256, *p* = 0.005).

Logistic regression was performed to evaluate the impact of prior nausea experience, nausea expectancy, age, diagnosis, state and trait anxiety, and global quality of life on the occurrence of post-treatment nausea (Table 5). The overall model was significant and explained 21% of the variance (Nagelkerke’s *R*^2^ = 0.21, χ^2^ = 19.2, *p* = 0.007). Prior nausea experience, lower age, and lower quality of life significantly predicted nausea occurrence (Table 5). After controlling for all other variables in the model, the odds to develop post-chemotherapy nausea were 2.6 fold higher in patients with prior nausea experience (*OR* = 2.592; 95% CI, 1.0 to 6.67; *p* < 0.05). Furthermore, the odds increased by 5% for each 1-year decrease in age (*OR* = 0.95; 95% CI, 0.92 to 0.99; *p* < 0.05) and by 3% for each 1-point decrease in global QoL (scale from 0 to 100; *OR* = 0.97; 95% CI, 0.94 to 1.0; *p* < 0.05). Diagnosis of breast cancer was associated with 2-fold higher odds compared to other types of cancer, but confidence intervals were large and the difference did not reach the level of significance (*OR* = 1.96; 95% CI, 0.91 to 1.03; *p* = 0.287). When controlling for all other variables including prior nausea experience, nausea expectancy scores did not predict nausea occurrence (*OR* = 1.014; 95% CI, 0.51 to 2.02; *p* = 0.969) (Table 5).

**Table 5:** Binary regression analysis for occurrence of post-treatment nausea.

Predictor	b	SE	Wald	*P*-value	OR [95% CI]
Nausea expectancy score	0.014	0.352	0.002	0.969	1.014 [0.509; 2.02]
Nausea in other situations (no^∗^/yes)	0.953	0.482	3.898	0.048	2.592 [1.007; 6.674]
Age	−0.047	0.020	5.719	0.017	0.954 [0.918; 0.992]
Diagnosis (breast cancer^∗^/other cancer)	0.673	0.632	1.134	0.287	1.961 [0.568; 6.768]
State anxiety (STAI-State)	−0.030	0.032	0.882	0.348	0.971 [0.912; 1.033]
Trait anxiety (STAI-Trait)	0.033	0.036	0.810	0.368	1.033 [0.962; 1.109]
Global quality of life (EORTC-QLQ-C30)	−0.031	0.014	4.652	0.031	0.970 [0.943; 0.997]

*SE, standard error; OR, Odds ratio; EORTC-QLQ-C30, European Organization for Research and Treatment of Cancer-Quality of Life Questionnaire-C30; STAI, State-Trait-Anxiety Inventory. ^∗^Reference category figures.*

## Discussion

In this prospective cohort study, we investigated factors contributing to nausea expectation and post-treatment nausea in first-time chemotherapy patients with cancer. Results revealed that higher state anxiety and a positive history of nausea in other situations predicted the expectancy of nausea as a side effect of chemotherapy. Furthermore, high nausea expectancy levels and a positive history of nausea were associated with a higher risk to develop post-chemotherapy nausea. When controlling for medical, demographic and psychosocial variables in multiple regression analyses, however, only younger age, lower quality of life, and a positive history of nausea in other situations were confirmed as independent predictors.

In order to reduce sample heterogeneity, we included only patients receiving chemotherapeutic regimens with moderate emetogenic potential (Lohr, 2008). Since only 13 male patients could be recruited, we restricted our analyses to female patients. Even though all study patients received standard antiemetic drugs, 62% suffered from mild-to-moderate nausea during the 4 days following chemotherapy, emphasizing the need for improving nausea control in this patient group. 42% of our female cohort had a positive history of nausea in other situations, which appears to be above average. An epidemiologic study of more than 18.000 United States inhabitants reported a lifetime prevalence of nausea equaling 14.3% in females and 6.0% in males (Walker et al., 1992). A large population based study of more than 62.000 people living in Norway found that 12.5% of the population complained of nausea during the last year, with rates almost three times higher in females than in males (Haug et al., 2002). However, little information on the prevalence of nausea in the general population is available.

Our finding that more anxious patients developed higher nausea expectancies is in accordance with previous results indicating a close relationship between emotional distress and anticipated nausea in a comparable patient cohort (Montgomery and Bovbjerg, 2003; Colagiuri et al., 2008). In contrast to Colagiuri et al. (2008), however, we could not confirm a relationship between pretreatment quality of life and nausea expectancy. This may be due to differences in sample size (671 vs. 121 patients), the proportion of females (96% vs. 100%), and/or the assessment prior nausea experience (general lifetime past experience with nausea vs. motion sickness susceptibility). Remarkably, positive lifetime history of nausea was a strong predictor for nausea expectancy in our study. This finding is in line with previous studies (Carey and Burish, 1988) and supports Rotter’s social learning theory, which claims that expectancies are based on general lifetime experience in similar situations when a situation is new (Montgomery and Bovbjerg, 2003).

In agreement with Colagiuri et al. (2008), our results revealed that patients with high levels of nausea expectation showed higher post-chemotherapy nausea than patients with lower levels of expectancy (Table 4). In contrast to their results (Colagiuri et al., 2008), however, our bivariate analyses revealed only a weak and non-significant correlation between nausea expectancy scores and post-chemotherapy nausea (*r_sp_* = 0.13). Similarly, when controlling for other factors known to contribute to post-chemotherapy nausea including prior nausea history (Dodd et al., 1996; Colagiuri et al., 2008; Lohr, 2008; Montgomery et al., 2010; Rha et al., 2016), nausea expectancy did not predict the occurrence of nausea (Table 5). This unexpected finding may partly be due to our small sample of only 121 patients and the restriction of our analyses to female patients, which decreased statistical power and most probably also the variance in the sample. We used the same expectancy score as Colagiuri et al. (2008) and also assessed nausea in the 4 days following chemotherapy in patients newly diagnosed with cancer. Differently to Colagiuri et al. (2008), we applied binary regression analysis to evaluate the link between expectancy and nausea when controlling for other contributing variables due to our significantly skewed nausea scores. An exploratory linear regression analysis with mean nausea scores as the dependent variable, however, basically confirmed our findings (results not shown). A further difference to the study by Colagiuri et al. (2008) concerned the assessment of nausea history: While we asked patients for their general lifetime past experience with nausea, Colagiuri et al. focused on motion sickness susceptibility. Possibly, some types of nausea experience are more predictive for post-chemotherapy nausea than others. For example, one early study reported that the history of nausea and vomiting with foods, but not with motion, anxiety, or pregnancy, was related to the occurrence of post-chemotherapy nausea (Jacobsen et al., 1988). In agreement with Colagiuri et al. (2008), our results revealed that younger age and lower quality of life were independent predictors for post-chemotherapy nausea. The odds of developing nausea increased by almost 50% for each 10 year decrease in age and by almost 30% for each 10 points lower global quality of life on a 100-point VAS scale. However, the greatest impact on post-chemotherapy nausea was revealed for the history of nausea in other situations: Patients with prior nausea experience showed 2.6 fold higher odds to develop post-chemotherapy nausea than patients without such experience. Our findings therefore suggest that the association between high nausea expectation and high nausea after first-time chemotherapy is not necessarily causal in nature. Rather, it could be based on the knowledge of patients about their susceptibility to nausea.

Several experimental studies indicate that informing patients about specific side effects of drugs can increase their occurrence (Silvestri et al., 2003; Mondaini et al., 2007; Jacobs et al., 2017). Conversely, the reduction of negative expectancies by psychological interventions is believed to prevent the occurrence of non-pharmacological side effects (Colagiuri et al., 2008; Quidde et al., 2018). The results of our study suggest that this may not necessarily apply to chemotherapy-induced nausea in the age of modern antiemetics. Rather, interventions that enhance quality of life may be more promising. This includes interventions that optimize positive treatment expectancies, which in turn increase quality of life (Rief et al., 2017). Quality of life could also be enhanced by non-pharmacological co-interventions such as acupuncture (Dean-Clower et al., 2010), interventions that are also known to induce large positive expectations (Ezzo et al., 2006; Linde et al., 2010; Meissner et al., 2013). Especially if patients are young, have a positive history of nausea in other situations, and/or present with reduced quality of life, such co-interventions should be considered in addition to antiemetic standard drugs. Reducing nausea during the first chemotherapy cycle could then also lower the risk to develop nausea before and during subsequent chemotherapy cycles (Andrykowski, 1990; Hickok et al., 2001; Montgomery et al., 2010).

Several limitations of our study need to be mentioned. Even though we restricted our sample to patients receiving moderately emetogenic chemotherapy regimens, we did not control for the different types of chemotherapeutic regimens and antiemetic drugs. This may have lowered the statistical power of our analyses. Furthermore, we restricted our analyses to female patients, most of whom suffered from breast cancer. The generalizability of our results to male patients and other types of cancer needs to be tested in future studies. In addition, despite the prospective design, our results cannot conclusively answer the question of causality between nausea expectancy and the occurrence of nausea after chemotherapy. Experimental designs that manipulate nausea expectancy before first-time chemotherapy will help to elucidate this issue (Quidde et al., 2018). Finally, asking patients about their expectancies to develop nausea might increase the occurrence of this side effect by eliciting a nocebo effect. However, a recent experimental study did not show enhanced nausea when expectancies of side-effects were assessed (Colagiuri et al., 2013).

Taken together, our results indicate that in addition to younger age and lower quality of life, prior experience with nausea is an important source of post-treatment nausea in first-time chemotherapy patients. Physicians should consider to assess these factors prior to the first chemotherapy to estimate the risk of post-chemotherapy nausea. In addition to antiemetic drugs, non-pharmacological co-interventions known to enhance quality of life, such as acupuncture, could help to reduce nausea and improve adherence to chemotherapy in very susceptible patients.

## Ethics Statement

The study protocol was approved by the ethical committee of the Medical Faculty at the Ludwig-Maximilians-University Munich, Munich, Germany (Ref. No. 259-13). All participants provided written informed consent.

## Author Contributions

KM and RF designed the study. NT and RF collected the data. KM, NT, and RF analyzed the data. KM drafted the first version of the manuscript. All authors contributed to the interpretation of the data and critically reviewed the manuscript.

## Conflict of Interest Statement

The authors declare that the research was conducted in the absence of any commercial or financial relationships that could be construed as a potential conflict of interest.
